# Relationship between coarse F waves and thromboembolic events in patients with permanent atrial fibrillation

**DOI:** 10.1002/joa3.12430

**Published:** 2020-09-02

**Authors:** Yahya Kemal İçen, Hasan Koca, Hilmi Erdem Sümbül, Arafat Yıldırım, Fadime Koca, Abdullah Yıldırım, Mustafa Lutfullah Ardıc, Mükremin Coşkun, Mehmet Uğurlu, Mevlüt Koç

**Affiliations:** ^1^ Department of Cardiology University of Health Sciences ‐ Adana Health Practice and Research Center Adana Turkey; ^2^ Department of Internal Medicine University of Health Sciences ‐ Adana Health Practice and Research Center Adana Turkey; ^3^ Göksun State Hospital Cardiology Department Kahramanmaraş Turkey

**Keywords:** atrial fibrillation, electrocardiography, left atrium, morbidity, thromboembolism

## Abstract

**Background:**

The coarse F waves on the 12‐lead surface electrocardiogram (ECG) in patients with atrial fibrillation (AF) are known as atrial viability and contractility indicator. Our aim in this study was to investigate the effect of coarse F wave on thromboembolism in patients with permanent AF.

**Methods:**

In our study, 328 patients with permanent AF were included. Routine laboratory, echocardiographic and electrocardiographic parameters were examined. Cerebrovascular event (CVE) or acute artery occlusion was considered a thromboembolic event.

**Results:**

In our study, 46 (14.0%) of the patients were found to have thromboembolic events and 282 (86%) of them were found without thromboembolic events. In the group with thromboembolic event, the number of patients with hypertension (HT) (*P* < .001) and history of coronary artery disease (*P* = .003) and elderly patients (*P* < .001) was significantly higher and warfarin use was significantly lower (*P* = .025). In the group of patients without thromboembolic events, the number of patients with a coarse F wave in surface ECG was significantly lower (*P* = .001). Age (OR: 1.105, 95% CI: 1.066‐1.145, *P* < .001), HT (OR: 2.831, 95% CI: 1.266‐6.331, *P* = .011), and coarse F wave (OR: 0.290, 95% CI: 0.126‐ 0.670, *P* = .004) were determined as independent variables for thromboembolic events.

**Conclusion:**

Coarse F wave in 12‐lead surface ECG in patients with permanent AF may be associated with good prognosis.

## Graphical abstract

The coarse F waves may be seen on electrocardiogram in permanent atrial fibrillation patients. This wave may be related to thromboembolism and morbidity.

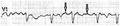

## 1 INTRODUCTION

Atrial fibrillation (AF), characterized by irregular R‐R intervals and absence of ‘p 'wave in 12‐lead surface electrocardiography (ECG), is still among the important causes of mortality and morbidity despite the developments in current treatment methods and the use of new generation drugs.^1^, ^2^, ^3^

In the classification made according to the current guideline, the beginning and ending style of the palpitation are taken into consideration. According to this, there are five groups; (a) first detected, (b) paroxysmal, (c) persistent, (d) long‐term persistent, and (e) permanent AF.^1^

Among these types, p wave is not observed in surface ECG in patients with permanent AF, instead, fibrillatory waves are observed.^4^, ^5^ If the atrial tissue is still able to maintain its viability, it is said that coarse F waves can be seen in surface ECG as a reflection of atrial contraction.^5^

In patients with atrial fibrillation, the left atrium (LA) and left atrial appendix (LAA) have been shown as the main source of thromboembolism.^4^ In the literature, left atrial size and left atrial appendix functions are closely associated with coarse F waves.^4^, ^5^, ^6^, ^7^ There are contradictory results related to thromboembolism in studies evaluating patients with AF with and without coarse F wave. In some studies, it has been mentioned that thromboembolic complications are less common in patients with coarse F waves,^7^, ^8^ while in some studies it has been reported that they may be unrelated or these complications are detected more in these patients.^4^, ^6^, ^9^, ^10^

According to our opinion, since p wave will not be detected in ECG in patients with permanent AF, coarse F waves, which are the indicators of viability and contractility in the atrium, become more important on thromboembolic events compared to other types of AF. For this reason, we hypothesized that it will be a more accurate approach to investigate the effect of coarse F waves on morbidity in patients with permanent AF.

In our study we aimed to investigate the effect of coarse F wave on thromboembolic events in patients with permanent AF.

## 2 METHODS

### 2.1 Study population

In our study, 511 patients with permanent AF who applied to our arrhythmia outpatient clinic between January 2015 and December 2019 were retrospectively screened. Since AF patients with heart failure and mitral valve stenosis tend to have thromboembolism, we thought that the net effect of coarse F waves could not be evaluated and we did not include these patients in the study. We excluded 183 patients from the study because hospital records of 85 patients were insufficient (ECG, laboratory tests, or follow‐up information), 24 patients had mitral valve stenosis, 34 patients had hemorrhagic cerebrovascular events (CVE), 30 patients had heart failure (HF), and 10 patients had kidney and liver failure. A total of 328 patients with permanent AF were included in the study. Demographic data and drug using were recorded. Dabigatran 110 or 150 mg 2 × 1, rivaroxaban 15 or 20 mg 1 × 1, edoxaban 30 or 60 mg 1 × 1, and apixaban 2.5 or 5 mg 2 × 1 were considered as new oral anticoagulant (NOAC). CHA_2_DS_2_‐VASc (congestive heart failure, hypertension, age ≥ 75 years, diabetes, stroke, vascular disease, age 65‐74 years, and gender category) score was calculated for all patients.^11^

### 2.2 Evaluation of laboratory findings

Renal function tests, lipid parameters, high sensitive CRP (hs‐CRP), uric acid, thyroid function tests, prothrombin time–international normalized ratio (PT‐INR), and complete blood count results were recorded from routine blood tests.

### 2.3 Electrocardiographic evaluation

**FIGURE 1:**
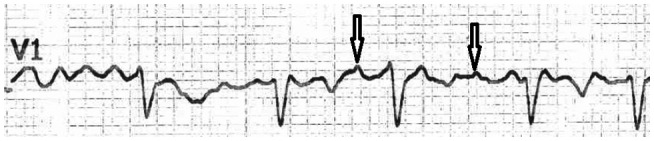
Showing of the coarse F wave records in lead V1 (arrows)

**FIGURE 2:**
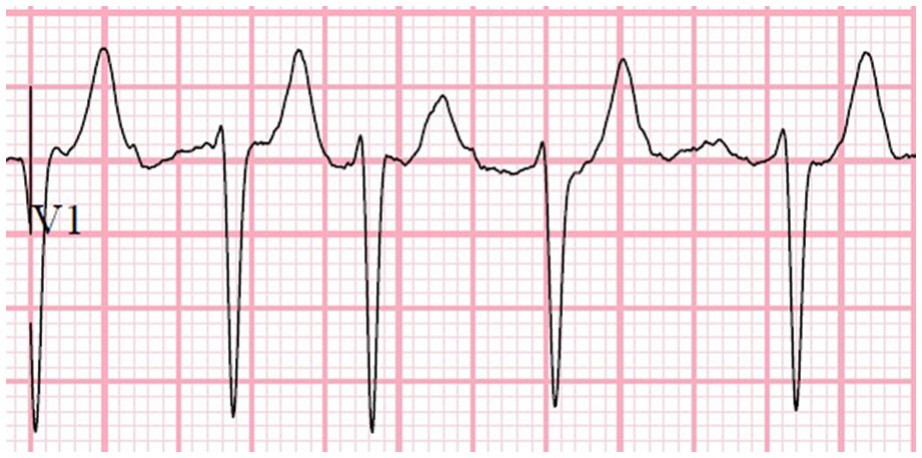
Showing of the electrocardiography example without a coarse F wave

The 12‐lead surface ECGs of the patients (Nihon Kohden, Cardiofax V; model ECG‐1550K, 25mm/sec speed and 1mv/ 10mm standard) were evaluated independently by two cardiologists (YKI and HK). Fibrillatory waves with an amplitude ≥ 0.5 mm in lead V1 was considered as the coarse F wave. ^5^ T wave or U wave was carefully distinguished to avoid artifacts (Figures 1 and 2).

### 2.4 Echocardiographic evaluation

From echocardiographic (Phillips Healthcare, DA Best, Netherlands) data, ejection fraction (EF), left ventricular diastole and end‐systolic diameters (LVDD, LVDS), and left atrium diameter (LAD) were recorded.

### 2.5 Thromboembolic event evaluation

Embolism or thrombus caused by atherosclerosis in large arteries, cardiac‐induced embolism, and small vessel occlusion (lacune) were accepted as embolic events.^12^ From the hospital records; Thromboembolic event evaluated by computed brain tomography (BBT), magnetic resonance imaging (MRI), color doppler ultrasonogography (RDUS), or physical examination findings.

### 2.6 Statistical analysis

The variables were divided into two categories, categorical and continuous. Categorical data were shown as numbers and percentages and compared with the chi‐square test. Whether continuous variables show normal distribution was calculated by the Kolmogrov–Smirnov test. Continuous variables were shown with mean and standard deviation. Normally distributed continuous variables were compared with independent simple *T* test, while non‐normally distributed variables were compared with Man–Whitney *U* test. Binominal logistic regression analysis was performed with the variables *P* < .05 and independent predictors for tromoembolic events were determined. Statistics were made in SPSS 20.0 (SPSS Inc, Chicago, IL, United States) in Windows operating system, *P* < .05 was considered statistically significant.

## 3 RESULTS

According to our results, 46 (14.0%) of the patients were detected with thromboembolic events and 282 (86%) of them without thromboembolic events. Among the patients with thromboembolic events, 4 (1.2%) had acute artery occlusion, others were CVE. When the demographic data are compared between the two groups; in the group of patients with thromboembolic events, the mean age (*P* < .001) and the number of patients with hypertension (*P* < .001) and a history of CAD (*P* = .003) were significantly higher, follow‐up durations (5.5 ± 0.9 vs 4.6 ± 2.1 years, *P* = .156) and other findings were similar (Table 1). When the pharmacological treatment used by the patients were compared, the number of patients using warfarin was significantly lower in the group of patients with thromboembolic events (*P* = .025), other pharmacological treatment were similar (Table 2). When the laboratory parameters of both groups were compared, there was no significant difference (Table 3). When electrocardiographic and echocardiogarphic data were compared, the number of patients with coarse F wave in surface ECG was significantly lower in the group of patients with thromboembolic events (*P* = .001), other findings were similar (Table 4). In binominal logistic regression analysis with significant parameters, age (OR: 1.105, 95% CI: 1.066‐1.145, *P* < .001), HT (OR: 2.831, 95% CI: 1.266‐6.331, *P* = .011), and coarse F wave (OR: 0.290, 95% CI: 0.126‐0.670, *P* = .004) were determined as independent predictors for thromboembolic events (Table 5). When 144 patients with coarse F waves (43.9%) and 184 patients without it were compared, the number of patients with hypertension, hyperlipidemia, and thrombembolic events was higher in the group without the coarse F wave (*P* = .004, *P* = .005, and *P* = .001, respectively), left atrium size increased significantly (*P* = .001), and other findings were similar (Table 6).

**TABLE 1:** Comparison of patients’ demographic findings

Age (yr)	72.3 ± 8.5	60.7 ± 10.8	<0.001
Male gender, n (%)	18 (39.1)	153(54.3)	0.057
Systolic blood pressure (mm Hg)	122.5 ± 18.9	124.8 ± 18.7	0.446
Diastolic blood pressure (mm Hg)	77.9 ± 15.0	80.7 ± 11.2	0.232
BMI (kg/m^2^)	27.2 ± 6.1	27.4 ± 7.7	0.801
Smoking, n (%)	10 (21.7)	81 (28.7)	0.327
DM, n (%)	19 (41.3)	79 (28.0)	0.068
HT, n (%)	36 (78.3)	135 (47.9)	<0.001
HPL, n (%)	16 (34.8)	51 (18.1)	0.009
CAD, n (%)	9 (19.6)	18 (6.4)	0.003
AF duration, n (yr)	5.5 ± 0.9	4.6 ± 2.1	0.1
CHA₂DS₂‐VASc score, n	3.5 ± 1.4	3.1 ± 0.9	0.377

Abbreviations: AF, atrial fibrillation; BMI, body mass index; CAD, coronary artery disease; CHA₂DS₂‐VASc, congestive heart failure, hypertension, age, diabetes mellitus, stroke, vascular disease, gender category; DM, diabetes mellitus; HT, hypertension; HPL, hyperlipidemia.

**TABLE 2:** Comparison of patients’ medications

	Patients with thromboembolic events (n = 46)	Patients without thromboembolic events (n = 282)	*P*
Warfarine (n, %)	21 (45.7)	178 (63.1)	.025
NOAC (n, %)	13 (28.3)	80 (28.4)	.988
ACE‐ARB (n, %)	16 (34.8)	128 (45.4)	.179
Calcium channel blocker (n, %)	6 (13.0)	40 (14.2)	.836
Β blocker (n, %)	35 (76.1)	183 (64.9)	.136
Furosemid (n, %)	5 (10.9)	35 (12.4)	.767
Amiodarone (n, %)	7 (15.2)	57 (20.2)	.428
Statin (n, %)	7 (15.2)	47 (16.7)	.806
Digoxin (n, %)	14 (30.4)	74 (26.2)	.552
ASA (n, %)	23 (50.0)	169 (59.9)	.205

Abbreviations: ACE, angiotensin converting enzyme; ARB, angiotensin receptor blocker; ASA, acetylsalicylic acid; NOAC, Novel oral anticoagulant.

**TABLE 3:** Comparison of patients’ laboratory findings

	Patients with thromboembolic events (n = 46)	Patients without thromboembolic events (n = 282)	*P*
WBC (uL)	7.2 ± 2.3	7.4 ± 2.6	.604
Hemoglobin (mg/dL)	13.6 ± 2.3	13.5 ± 1.9	.885
BUN (mg/dL)	31.5 ± 10.3	32.3 ± 21.5	.798
Cr (mg/dL)	0.8 ± 0.3	0.8 ± 0.2	.254
Na (mmol/L)	138.1 ± 3.1	138.7 ± 3.1	.138
K (mmol/L)	4.4 ± 0.5	4.2 ± 0.5	.078
Total cholesterol (mg/dL)	189.5 ± 42.5	183.9 ± 36.4	.409
LDL (mg/dL)	128.6 ± 31.1	126.5 ± 35.7	.731
HDL (mg/dL)	40.4 ± 9.9	42.2 ± 12.5	.399
Triglyceride (mg/dL)	160.1 ± 70.9	178.8 ± 92.9	.268
Hs‐CRP (mg/L)	3.4 ± 3.6	2.3 ± 2.8	.065
Uric acid (mg/dL)	7.1 ± 2.5	6.9 ± 2.2	.615
T4 (ng/dL)	1.3 ± 0.2	1.3 ± 0.3	.957
TSH (uIU/dL)	1.8 ± 1.4	1.9 ± 1.5	.752
PT‐INR, (n)	2.6 ± 0.8	2.7 ± 0.7	.859

Abbreviations: BUN, blood urea nitrogen; Cr, creatinin; HDL, high‐density lipoprotein; Hs‐CRP, high‐sensitive C‐reactive protein; Htc, hematocrit; LDL, low‐density lipoprotein; PT‐INR, prothrombin time–international normalized ratio; TSH, thyroid stimulation hormone; WBC, white blood cells.

**TABLE 4:** Comparison of patients’ electrocardiographic and echocardiographic findings

	Patients with thromboembolic events (n = 46)	Patients without thromboembolic events (n = 282)	*P*
Coarse F wave, n (%)	10 (21.7)	134 (47.5)	.001
EF (%)	57.7 ± 6.5	56.9 ± 6.3	.693
LVDD (mm)	49.8 ± 3.6	47.2 ± 4.6	.084
LVSD (mm)	29.9 ± 5.1	30. 7 ± 3.8	.490
Left atrial diameter, (mm)	50.8 ± 5.6	48.8 ± 5.3	.092

Abbreviations: EF, ejection fraction; LVDD, left ventricule end‐diastolic diameter; LVDS, left ventricule end‐sistolic diameter.

**TABLE 5:** Independent predictors for thromboembolic events

	Odds ratio	95% confidence interval	*P*
Age	1.105	1.066‐1.145	<.001
HPL	0.689	0.278‐1.704	.689
HT	2.831	1.266‐6.331	.011
HPL	1.452	0.587‐3.593	.420
CAD	2.430	0.904‐6.530	.078
Warfarin	0.609	0.282‐1.315	.207
Coarse F wave	0.290	0.126‐0.670	.004

Abbreviations: CAD, coronary artery disease; DM, diabetes mellitus; HT, hypertension; HPL, hyperlipidemia.

**TABLE 6:** Comparison of the findings of patients with and without coarse F wave

	Patients with coarse F waves (n = 144)	Patients without coarse F waves (n = 184)	*P*
Age (yr)	61.9 ± 11.2	62.7 ± 11.3	.553
Male gender, n,(%)	81 (56.2)	90 (48.9)	.187
Systolic blood pressure (mm Hg)	120.7 ± 19.6	124.6 ± 16.7	.112
Diastolic blood pressure (mm Hg)	74.9 ± 16.4	76.9 ± 12.3	.232
BMI (kg/m^2^)	24.2 ± 3.1	26.1 ± 3.7	.441
Smoking, n (%)	38 (26.4)	53 (28.8)	.628
DM, n (%)	42 (29.2)	56 (30.4)	.803
HT, n (%)	62 (43.1)	109 (59.2)	.004
HPL, n (%)	18 (12.5)	46 (25.0)	.005
CAD, n (%)	8 (5.6)	19 (10.3)	.119
AF duration, n (yr)	3.8 ± 1.6	4.4 ± 2.1	.081
CHA₂DS₂‐VASc score, n	3.1 ± 1.6	2.9 ± 1.1	.385
Presence of thromboembolic event, n	10 (6.9)	36 (19.6)	.001
PT‐INR, (n)	2.4 ± 1.1	2.9 ± 0.6	.552
EF (%)	56.3 ± 6.8	58.1 ± 6.1	.340
LVDD (mm)	47.2 ± 4.5	47.4 ± 4.8	.653
LVSD (mm)	30.4 ± 3.7	30.9 ± 4.1	.457
Left atrial diameter, (mm)	47.5 ± 5.2	51.4 ± 5.3	.001

Abbreviations: AF, atrial fibrillation; BMI, body mass index; CAD, coronary artery disease; CHA₂DS₂‐VASc, congestive heart failure, hypertension, age, diabetes mellitus, stroke, vascular disease, gender category; DM, diabetes mellitus; EF, ejection fraction; LVDD, left ventricule end‐diastolic diameter; LVDS, left ventricule end‐sistolic diameter; HT, hypertension; HPL, hyperlipidemia; PT‐INR, prothrombin time–international normalized ratio.

## 4 DISCUSSION

In our study where we investigated the effect of coarse F wave on thromboembolic events in surface ECG in patients with permanent AF, we found several important results. The most important of these is that thromboembolic event appears less in patients with a coarse F wave. In this study, patients with heart failure and mitral valve stenosis, which are directly related to thromboembolic events, were excluded, and the relationship between the coarse F wave and thromboembolic events was clearly determined. In addition, thromboembolic events were found to be closely related to age and HT.

Compared to other types of AF, patients with permanent AF usually do not show p waves showing atrial contraction at all, instead thin fibrillatory waves may appear in surface ECG.^10^ In some patients, the amplitude of these fibrillatory waves is >0.1 mm, which is called the coarse F wave.^5^ In our opinion, in these patients who have been diagnosed with AF for a long time, coarse F waves, which are an indicator of the ability of viable atrium tissue to contract, may cause a shaking by causing contraction in the left atrium and left atrial appendix, which is the main source of thrombogenicity. Thus, one or more beats cause blood flow and venous stasis may decrease in LA and LAA. In previous studies, the results supporting our opinion were obtained.^4^, ^5^ There are also studies suggesting that coarse F waves may reflect atrial hypertrophy and increased thromboembolic event.^10^, ^13^ Owing to the reduction in venous stasis in the left atrium and LAA, thrombogenicity has decreased and therefore, in our study, patients with coarse F wave may have seen less thromboembolic events.

Nakagawa et al investigated hemostatic abnormality and LAA dysfunction with fibrillatory wave amplitude in patients with chronic non‐rheumatic AF.^8^ In this study, it was stated that cerebral embolism was found less in patients with a coarse F wave. It is also stated that fine fibrillatory waves in V1 lead are a useful parameter that can be used in LAA dysfunction. Although the results of this study are compatible with the results of our study, LAA was not evaluated with transesophageal echocardiography (TEE) in our study. In our opinion, since conversion to sinus rhythm is not considered in patients with permanent AF, evaluating patients with TEE will not be a very beneficial approach or there may be risks. In addition, it will be an expected condition to detect thrombus in LAA or LA in patients with permanent AF. For this reason, we think that routine transthoracic echocardiographic (TTE) evaluation may be sufficient in these patients if there is no other indication.

Another study by Mutlu et al stated that in patients with rheumatic mitral stenosis, coarse F waves are a risk factor for thromboembolic events.^9^ In the same study, it was also stated that the LA and LAA of the patients with coarse F waves tend to expand, but the functions of these chambers are not related to the coarse F waves. The greatest disadvantage of this study, whose results are contrary to our study, is that it was performed in patients with mitral stenosis. With the increase in left atrial pressure in patients with mitral stenosis, the enlargement of the left atrium will be inevitable and the frequency of thromboembolic events will increase. Therefore, thromboembolism will appear more frequently with increasing number of patients. According to us, Mutlu et al may have made such an association since they included more patients with coarse‐wave AF. In our study, patients with mitral stenosis and heart failure were excluded from the study because we thought that it may cause complexity in the effects of coarse‐wave AF. In another study in which fibrillatory wave amplitudes were evaluated clinically in patients with persistent AF, it was emphasized that patients with coarse F waves were younger and had a shorter follow‐up period.^14^ The decrease in the frequency of the coarse F waves with age coincides with our study, but the follow‐up times were similar in our study between the two groups. There are studies mentioning that coarse F wave can be used in both relapse and long‐term follow‐up in patients with persistent AF who underwent catheter ablation.^15^, ^16^ In a study by Cheng et al,^15^ in which cardioversion (CV) success and AF progression were examined, high‐amplitude fibrillatory waves were associated with unsuccessful CV and poor progression. Blackshear et al stated that the LAA function and velocity are not correlated with the coarse F waves because they cannot simultaneously evaluate coarse F waves and LAA functions.^18^ There are also drug studies that mention that antiarrhythmic drugs such as procainamide, ibutilide, flecainide, and amiodarone reduce fibrillatory waves by slowing intraatrial conduction.^17^, ^18^ In our study, such an effect cannot be mentioned since the number of patients using antiarrhythmic drug (amiodarone) is similar.

In addition, in our study, warfarin use was found to be less associated with thromboembolic events in univariate analyzes, and also could not be detected as an independent marker in multivariate analysis. In a retrospective study by Shpak et al,^19^ it was stated that more ischemic events were detected in patients using NOAC. In another large‐scale retrospective study, it was stated that no significant difference was found between NOAC and warfarin use in terms of ischemic events and embolism.^20^ It was observed that warfarin and NOAC use were similar in terms of thromboembolic events, consistent with previous studies.

### 4.1 Limitations

Since our study was retrospective, a maximum of 3‐4 surface ECG recordings in the patient file records could be examined. If longer continuous recordings could have been examined, the number of AF patients with coarse waves might have been found to be increased. Age and hypertension are known risk factors for thromboembolism. In patients with atrial fibrillation, it would be more logical to select individuals from similar age and hypertensive patient groups, to have a stronger claim for that coarse F waves are protective from thromboembolism. Since our patients are generally >60 years old, the presence of atheroma plaques in the carotid or vertebral arteries as a source of thromboembolism has not been examined. Some of our patients may have had thromboembolism for this reason. In addition, according to multivariate analysis, many known parameters associated with thromboembolic events associated with coarse F wave may have caused overfitting.

## 5 CONCLUSION

The coarse F waves in surface ECG in permanent AF patients may be a sign of good prognosis in terms of thromboembolic events. If coarse F waves are absent on the surface ECG of the patient, the patient should be followed closely and more attention should be paid for anticoagulation. If these patients were using coumadin, PT‐INR monitoring should be done more closely.

## DISCLOSURE

The authors have not conflicts of interest. The study was approved by Adana City Hospital Institutional Review Board, number 467, and date 22.05.2019.
